# Supplementary material for: Predicting visual function by interpreting a neuronal wiring diagram
Source: Nature. 2024 Oct 2;634(8032):113–23. doi: 10.1038/s41586-024-07953-5 (PMC11446822; doi:10.1038/s41586-024-07953-5)
Supplement: Supplementary file 6 — CRF and ERF predictions for individual TmY4 and TmY9 cells. Analogous to Supplementary Data 3, but for TmY target types. Shown are the top four monosynaptic pathways, the strongest pathway passing through each of the top ten intermediary types (ranking from Extended Data Fig. 7), and the trisynaptic pathway Tm1–TmY–Dm3–TmY (see the section entitled Prediction of spatial normalization). [file 41586_2024_7953_MOESM6_ESM.zip › DataS4/TmY4/720575940626158461.pdf]

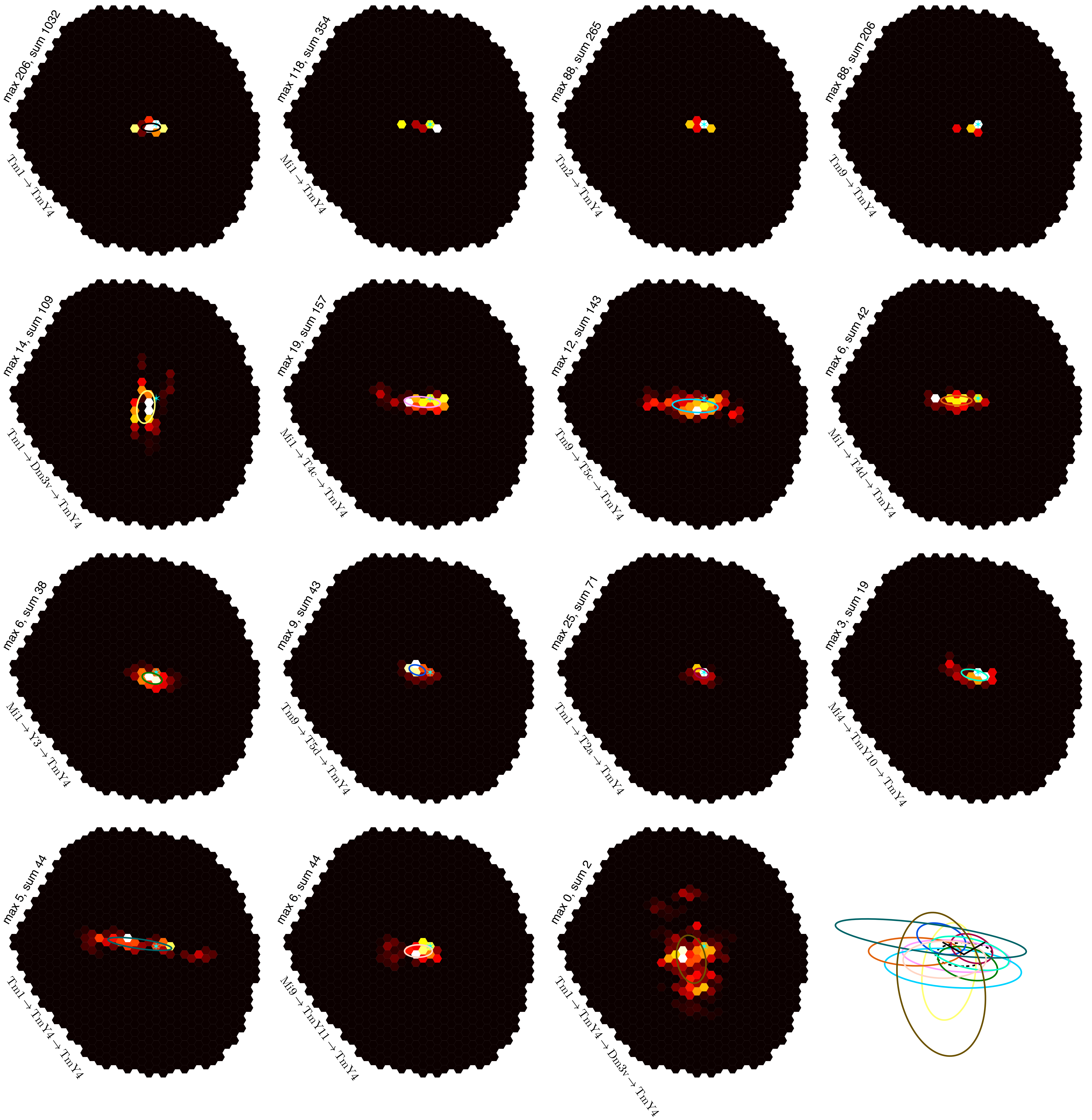
max 206, sum 1032
Tm1 → TmY4
max 118, sum 354
Mi1 → TmY4
max 88, sum 265
Tm2 → TmY4
max 88, sum 206
Tm9 → TmY4
max 14, sum 109
Tm1 → Dm3v → TmY4
max 19, sum 157
Mi1 → T4c → TmY4
max 12, sum 143
Tm9 → T5c → TmY4
max 6, sum 42
Mi1 → T4d → TmY4
max 6, sum 38
Mi1 → Y3 → TmY4
max 9, sum 43
Tm9 → T5d → TmY4
max 25, sum 71
Tm1 → T2a → TmY4
max 3, sum 19
Mi4 → TmY10 → TmY4
max 5, sum 44
Tm1 → TmY4 → TmY4
max 6, sum 44
Mi9 → TmY11 → TmY4
max 0, sum 2
Tm1 → TmY4 → Dm3v → TmY4
